# Modulation of Astrocyte Activity by Cannabidiol, a Nonpsychoactive Cannabinoid

**DOI:** 10.3390/ijms18081669

**Published:** 2017-07-31

**Authors:** Ewa Kozela, Ana Juknat, Zvi Vogel

**Affiliations:** 1 The Dr Miriam and Sheldon G. Adelson Center for the Biology of Addictive Diseases, Sackler Faculty of Medicine, Tel Aviv University, 6997801 Tel Aviv, Israel; ana.juknat@weizmann.ac.il (A.J.); zvi.vogel@weizmann.ac.il (Z.V.); 2 Neurobiology Department, Weizmann Institute of Science, 76100 Rehovot, Israel

**Keywords:** cannabidiol, cannabinoid, astrocyte, neurodegeneration, neuroinflammation

## Abstract

The astrocytes have gained in recent decades an enormous interest as a potential target for neurotherapies, due to their essential and pleiotropic roles in brain physiology and pathology. Their precise regulation is still far from understood, although several candidate molecules/systems arise as promising targets for astrocyte-mediated neuroregulation and/or neuroprotection. The cannabinoid system and its ligands have been shown to interact and affect activities of astrocytes. Cannabidiol (CBD) is the main non-psychotomimetic cannabinoid derived from *Cannabis*. CBD is devoid of direct CB1 and CB2 receptor activity, but exerts a number of important effects in the brain. Here, we attempt to sum up the current findings on the effects of CBD on astrocyte activity, and in this way on central nervous system (CNS) functions, across various tested models and neuropathologies. The collected data shows that increased astrocyte activity is suppressed in the presence of CBD in models of ischemia, Alzheimer-like and Multiple-Sclerosis-like neurodegenerations, sciatic nerve injury, epilepsy, and schizophrenia. Moreover, CBD has been shown to decrease proinflammatory functions and signaling in astrocytes.

## 1. Astrocytes

Astrocytes are the most numerous cells populating the central nervous system (CNS). Research during the last two decades has shown that they actively participate in brain development and activity, and maintain tissue homeostasis, far beyond their previously believed role of a sole “glue”-like support for neurons. In healthy brain, astrocytes regulate neuronal growth and synapse formation and pruning, support neuro-signaling (e.g., by forming the so called “tri-partite synapse”), regulate glutamate, potassium, and calcium release and uptake, and modulate synaptic potentiation and learning [1,2]. Astrocytes serve as the primary glycogen storage site in the CNS, and they perform metabolite cleansing. Together with endothelial cells, they form the blood brain barrier (BBB) providing selective protection to the brain.

Astrocytes, together with microglia, are the primary mediators of “reactive gliosis”, defined as the reactive response of glial cells to various CNS insults such as infection, trauma, ischemia, and neurodegenerative diseases. Reactive gliosis involves phenotypic and molecular changes of astrocytes and of microglia, and frequently leads to scar formation, an indicator of many brain injuries and pathologies. Glial scars may have a protective function by limiting the spread of neurodegeneration and infection as well as of ischemia-induced cellular rearrangements. However, more severe threats, such as autoimmune neuroinflammation and T cell/monocytes infiltration, may lead to a higher degree of astrogliosis, thus changing the tissue structure and damaging neuronal connectivity.

Astrocytes are crucial regulators of innate and adaptive immune responses in the CNS. Astrocytes participate in antigen recognition. Following their activation, the astrocytes secrete chemokines, cytokines, neurotoxic factors, molecules affecting blood vessel diameter (thus modulating blood flow), such as nitric oxide and prostaglandins, and by this way affect BBB permeability.

## 2. Astrocytes as Potential Therapeutic Target. Role of Cannabinoid System

Due to their crucial involvement in brain function, astrocytes have gained robust interest as a potential target for neurotherapies. Astrocyte activity has been shown to be involved in acute injury, neurodegeneration; to affect neuroplasticity, learning and memory; and to accompany neuropsychiatric disorders, drug addiction and dependence [3,4]. An accumulation of oxidative stress and chronic neuroinflammation as a result of inefficient astrocytic clearance of debris or misfolded proteins has been observed to significantly contribute to neuronal degeneration, progressing cognitive loss and motor disability. Thus, the pharmacological modulation of reactive astrocytes has been proposed as a tool to blunt neuronal damage and to slow the course of brain diseases [5,6].

The precise regulation of astrocyte activity is still far from understood, although a few candidate molecules/systems arise as promising targets for astrocyte-mediated neuroprotection. The endocannabinoid system and its ligands provide such a possibility. Mounting evidence shows that many cannabinoids, including phytocannabinoids (constituents of the *Cannabis* plant), endocannabinoids, and synthetic ligands, can modulate the intensity of reactive gliosis, resulting in neuromodulatory, anti-inflammatory, and neuroprotective effects in both mature and immature brain [7,8,9,10].

The cannabinoid system is comprised of two plasma membrane Gi/o-coupled cannabinoid receptors (CB1 and CB2) and a family of their endogenous ligands (*N*-arachidonoylethanolamine (anandamide) and 2-arachidonoylglycerol (2-AG)) together with synthesis/degradation enzymatic machinery (the serine hydrolases fatty acid amide hydrolase (FAAH) and monoacylglycerol lipase (MAGL), respectively). It has been shown that neurons express mostly CB1 cannabinoid receptors, while microglia express CB2. Interestingly, both types of the cannabinoid receptors were found to be present on astrocytes. Remarkably, CB2 expression was shown to be elevated in neuroinflammatory conditions in both microglia and astrocytes [11,12]. The expression of CB2 in neuroinflammatory conditions takes place in parallel with increased levels of endocannabinoids [13,14], further indicating the involvement of the cannabinoid system in the regulation of brain pathology and/or recovery.

*Cannabis* plant preparations (marijuana, hashish) are a source of over 100 cannabinoid chemicals with diverse affinities towards CB1 and CB2 receptors. The psychoactive material, Δ9-Tetrahydrocannabinol (THC, a CB1 and CB2 ligand), and the non-psychoactive Cannabidiol (CBD, binding neither CB1 nor CB2) are the most abundant phytocannabinoids in Cannabis, and exert a variety of bioactivities, including neuroprotective and anti-neuroinflammatory effects. Indeed, THC was shown to be neuroprotective (via CB1 mediated mechanisms) and to protect glial cells from apoptosis [15,16]. However, justified concerns have arisen regarding a possible deleterious effect of CB1 activation resulting in psychoactivity and memory disruption, particularly in the immature brain [17]. CB2 selective cannabinoids seem to serve as attractive therapeutics as they would presumably invoke minimal psychoactive responses. However, recent observations show heteromer CB1/CB2 functional interaction, suggesting that even CB2 ligands may induce some THC-like effects upon their activation [12,18]. Interestingly, cannabinoids devoid of both CB1 and CB2 activities (such as CBD) have also been shown to affect microglia and astrocyte functions (see below).

CBD, a major non-psychoactive constituent of *Cannabis*, has been described as neuroprotective, anti-oxidant, and anti-inflammatory in various models of brain insults (reviewed by [19,20,21,22,23]). In addition, CBD has been shown to be cytotoxic in cancer cells, to reduce pain, spasticity, and seizures, to reduce peripheral and CNS inflammation, to modulate blood circulation and metabolism, and to be anxiolytic and reduce psychosis [22,23,24]. There is increasing evidence that such broad CBD beneficial effects in the CNS involves the modulation of astrocyte activity, although the data are still scarce and fragmentary. Therefore, our aim in this manuscript is to summarize the findings on the effects of CBD on astrocytes in various experimental models of brain insults and pathologies.

## 3. Neurodegeneration

A vast number of preclinical studies demonstrate that CBD provides neuroprotection against acute and chronic brain injury across various developmental stages and species [21,22,24]. CBD was shown to be protective in cortical neuronal cultures against glutamate neurotoxicity [25]. It decreased the necrotic and apoptotic damage in forebrain slices in newborn mice and rats exposed to oxygen-glucose deprivation [26,27] and in the immature brain of piglets [28].

We still have a limited understanding of the mechanisms of CBD neuroprotection, although it is clear by now that the drug’s effects are not limited to the direct modulation of neurons, but are extended to microglia and astrocytes [10,15,29,30]. Decreasing the inflammatory responses of either microglia or astrocytes were shown to be a critical component of the neuroprotective process [31]. CBD is a potent regulator of oxidative stress, a leading cause of neurodegeneration, as it scavenges the reactive oxygen species (ROS) and reduces lipid peroxidation [30,32,33].

### 3.1. Stroke, Hypoxia-Ischemia

Stroke is among the most frequent causes of death and adult disability in developed countries. The majority of cerebral strokes are caused by the transient or permanent occlusion of a cerebral blood vessel, causing shortages in oxygen and glucose supplies to the brain tissue (“ischemic stroke”) and eventually leading to neuronal and other cell death (brain infarction/lesions). A hypoxia-ischemia episode (HI) is followed by a latent cell excitotoxicity, inflammation, and an increase in oxidative stress, as well as delayed energy failures, altogether causing secondary deterioration [34]. Cognitive impairments (memory, attention, executive function), neurological (motor disabilities) and behavioral dysfunctions are among the sequalae following brain HI.

An increase in astrocyte activity, characterized by increased cell proliferation (cell hypertrophy) and upregulation of the astrocyte intermediate filaments, including glial fibrillary acidic protein (GFAP) and vimentin [35], was shown to accompany almost every type of brain insult and injury, including focal and transient global brain ischemia [35,36,37]. Increased GFAP immunoreactivity in the CA1 hippocampal region after brain ischemia has been associated with the extent and maturation of neuronal necrosis [38,39]. Thus, decreasing astrogliosis may rescue neuronal damage and may serve as a valuable therapeutic strategy.

Although the efficiency of CBD in HI-related neurodegeneration has been intensively studied [21,22,40], only several reports have evaluated the effect of CBD on HI-induced astroglial activation. The effect of CBD was studied in adult mice with transient focal ischemia induced by middle cerebral artery (MCA) occlusion [41] as well as with transient global ischemia induced using bilateral common carotid artery occlusion (BCCAO; [42]). Immunohistochemistry revealed increased GFAP expression 3 days following the MCA procedure, and its reduction following 3 mg/kg of CBD administration [41]. Similarly, the global transient BCCAO-induced ischemia was accompanied by increased GFAP expression 7 days later, and CBD at 30 mg/kg reduced the GFAP expression [42]. The differences in the effective CBD doses used in these experiments may be related to the severity of the model used (focal vs. global ischemia). However, the different mice strains used could also account for these divergences in effective doses. The infarcts were characterized by excessive neuronal death and microglia activation (Iba1 staining) and resulted in neurological and motor impairments and memory disruption, all effectively reversed by CBD [41,42].

Young mice (9–10 days old) were used to evaluate the therapeutic time window of CBD administered at 1 mg/kg at various time points within 24 h after left common carotid artery electrocoagulation leading to transient hypoxia. GFAP expression was elevated 7 days after the procedure, and was reduced by 50% by CBD. CBD also reduced the HI-induced ipsilateral hemisphere volume loss, neuronal cell death, and microglial activation [43]. Interestingly, the CBD rescuing effect was seen only if the drug was administered up to 18 h following the HI procedure at all measured parameters including astrogliosis, ipsilateral hemisphere volume loss, and neuronal apoptosis [43].

Similarly, CBD was found to reduce GFAP levels in young rats (7–9 days old) that were increased following the MCAO procedure [44]. CBD also reduced neuronal death and microglia activation, and improved neurobehavioral losses following the insult. Interestingly, the effect on astrocyte activation was observed only at day 15 post HI, and not earlier at day 7 [44].

New-born piglets were used to evaluate the ability of CBD to reduce early life HI consequences [45]. CBD given either 15 min or 4 h after the procedure prevented HI-induced immediate brain damage, including neuronal as well as astrocyte swelling and death (indicated by reduced GFAP staining). In addition, astrocyte activation, estimated by using the serum content of S100β protein in cerebrospinal fluid, was increased in response to HI and diminished in the presence of CBD [45]. S100β is a calcium-binding protein produced mainly by the astrocytes. Although its role is not yet fully understood (it possibly acts as a growth factor in the developing brain), an increased level of S100β was shown to be associated with various brain injuries and stroke, and its raised levels were even proposed to serve as an early indicator of neurodegenerative diseases, including Alzheimer’s disease [46].

Hind and co-authors [47] used an in vitro model of the BBB, consisting of human astrocytes co-cultured with human brain microvascular endothelial cells and subjected to transient glucose and oxygen deprivation (OGD). CBD was observed to improve the BBB’s permeability in post-OGD co-cultures, which was accompanied by decreased cell damage and the reduced release of several neuroinflammatory molecules, including vascular cell adhesion molecule 1 (VCAM1), interleukin (IL)-6, and vascular endothelial growth factor (VEGF). The cellular source of the affected proinflammatory molecules, endothelial cells or astrocytes, was not defined [47].

### 3.2. Sciatic Nerve Transection

Sustained reactive astrogliosis may impair axonal regeneration [48]. Accordingly, genetic GFAP ablation in mice results in less dense scars and improved regeneration after a hemisection of the spinal cord, leading to improved survival and to the differentiation of neural progenitor-derived neurons and astrocytes [48,49].

Perez and coauthors [50] investigated the survival of dorsal root ganglia (DRG) and spinal motoneurons, the preservation of spinal cord circuits, and level of reactive gliosis following a unilateral sciatic nerve transection in 2-day neonatal rats in the presence or absence of CBD injected for 5 days following injury. Indeed, CBD injections into the injured mice resulted in a significant rescue of DRG neurons, spinal motoneurons, and pre-synaptic terminals, which was coupled with a reduction of neuronal apoptosis and of astrogliosis (based on GFAP immunoreactivity on day five post nerve transection).

Glutamate cytotoxicity is one of the leading causes of neuronal damage. Astrocytes are the main cells regulating glutamate synaptic levels, expressing glutamine synthetase (GS) as well as glutamate transporters, the glutamate transporter 1 (GLT-1) and glutamate aspartate transporter (GLAST). The levels of the latter have been shown to be decreased in brain insults [51]. Although CBD was shown to be protective against acute glutamate toxicity in cortical neurons [25], there is no data on the effect of CBD on glutamate metabolism/transporters in astrocytes.

## 4. Chronic Neurodegenerative Diseases

### 4.1. Alzheimer’s Disease

Alzheimer’s disease (AD) is the most common age-related neurodegenerative disorder. Its specific hallmarks are neurofibrillary tangles (deposition of hyperphosphorylated *tau* proteins) and senile plaques (extracellular lesions composed of β-amyloid (Aβ) aggregates) surrounded by activated astrocytes and dystrophic neuritis [52,53]. Moreover, an age-related increase in reactive gliosis was also correlated with Alzheimer-like cognitive declines [54].

Aβ neurotoxicity includes the production of ROS, changes in cytosolic calcium homeostasis, the activation of the glycogen synthase kinase 3 β (GSK3β) pathway (promoting amyloid precursor protein processing), and pro-inflammatory nuclear factor κ-light-chain-enhancer of activated B cells (NF-κB) cascade activation. CBD was found to inhibit many of these processes. As shown by Iuvone and co-authors [32], Aβ application onto rat pheocromocytoma PC12 cells caused significant cell death, which was reduced following CBD administration. Scavenging ROS, reduction of lipid peroxidation, proapoptotic caspase 3 activity, DNA fragmentation, and intracellular calcium levels were the main mechanisms suggested by the authors to be involved in CBD protective effects [32]. Moreover, CBD blunted *tau* hyperphosphorylation via reducing GSK3β phosphorylation, acting as a Wnt/β-catenin pathway rescuer [55] (reviewed in [56]).

The pharmacological inhibition of Aβ-induced reactive gliosis was proposed as a tool to blunt neuronal damage and to slow the course of AD [53]. Aβ inoculation into rat hippocampus led to a substantial neurodegeneration, accompanied by neuroinflammation and increased astrogliosis [57,58]. A 7- or 15-day administration of CBD (10 mg/kg) almost completely rescued the CA1 pyramidal neurons’ integrity, decreased iNOS and IL-1β levels, and downregulated GFAP immunostaining and S100β release. In addition, it was shown that CBD decreased GFAP and S100β levels, NF-κB pathway activation, and iNOS and IL-1β levels in Aβ-stimulated cultured newborn rat astrocytes [57,58].

### 4.2. Autoimmune Diseases

Multiple Sclerosis (MS) is an inflammatory demyelinating disease of the CNS, which is manifested by severe neurological and cognitive disabilities [59]. The disease is initiated by peripheral autoreactive T cells falsely primed against the body’s own myelin, this way causing the loss of neuronal connectivity and inducing neurodegeneration. In the experimental autoimmune encephalomyelitis (EAE, an animal model of MS), peripherally initiated inflammation impairs BBB permeability, allowing T cells’ and monocytes’ infiltration, and leading to microglia and astrocytes activation. Astrocytes play a key though diverse role in the MS-like neuroinflammation at all stages of the disease. Astrocytes are the main cells involved in the clearance of myelin and neuronal debris, scavenging excessive glutamate and ROS, and in this way reducing local cell damages. On the other hand, reactive astrocytes expressing high GFAP levels were shown to be a key source of CCL2, a chemokine involved in the recruitment of monocytes into the CNS, impairing BBB integrity and facilitating the influx of inflammatory modulators [60,61]. Astroglial scars, covering dysfunctional areas in the white matter, are considered to be hallmarks of the MS disease [62]. Interestingly, GFAP, the most common astrocyte marker, was apparently discovered and initially isolated from plaques of MS patients that consisted primarily of fibrous astrocytes and demyelinated axons [63]. Therefore, targeting astrocyte activation was proposed as an attractive therapeutic strategy in MS-like autoimmune neuroinflammatory pathologies, including in secondary progressive MS [61,64,65].

Various CB1/CB2 cannabinoid agonists have been shown to improve motor disabilities in EAE and to decrease T cell infiltration and neuroinflammation in the CNS [66,67,68,69]. Neuronal CB1 activation was shown to exert neuroprotective effects, while CB2 activation on T cells has been described to have a direct immunosuppressive effect [68]. Interestingly, we have shown that CBD (not binding CB1/CB2) ameliorates the clinical symptoms of myelin oligodendrocyte glycoprotein (MOG)35-55-immunized EAE mice, reducing their microglial activation and the leukocyte infiltration into the CNS. In addition, we showed that CBD reduces the activity of Th17 cells, a key T cell phenotype mediating MS-like pathologies [70,71,72,73]. CBD was confirmed to be neuroprotective and immunoregulatory in a Theiler’s murine encephalomyelitis virus-induced demyelinating disease (TMV-IDD), a viral model of MS [74]. In parallel, CBD decreased the spinal cord expression of CCL2 and CCL5 chemokines and the endothelial release of VCAM1, lessening leukocyte adherence [74].

Newborn rat astrocyte cultures were employed by Guaza’s group to evaluate the CBD effect in astrocytes. The IL-1β + TNFα-stimulated astrocytes released high levels of CCL2, which were decreased in the presence of CBD [74]. The same group studied the effects of the *Sativex*-like botanical mixture (containing THC, CBD, and several other *Cannabis* components) in the TMV-IDD mice model, observing improved motor abilities, decreased neuroinflammation, reduced astrogliosis, and reduced GFAP and vimentin staining [75]. Chondroitin sulfate proteoglycans (CSPGs) content was shown to be increased on the edges of MS astroglial scars [76], and was suggested to act as a potent inhibitor of axon regeneration [77]. The accumulation of CSPGs in the mouse viral model of EAE was decreased by the *Sativex-*like botanical extract [75]. It should be noted that this study does not define which component of the *Sativex* mixture (THC or CBD, both together or other cannabinoid components present, see [75] for details) is responsible for the observed decrease in astrocyte activity.

Very recently, a topical application of 1% CBD cream was shown to diminish EAE clinical disability scores in MOG35-55 immunized EAE mice. In parallel, it led to decreases in neuroinflammation, neuronal apoptosis, and GFAP expression, demonstrating the role of astrocytes in the process [78].

### 4.3. Huntington’s Disease

Huntington’s disease is an inherited neurodegenerative disorder that leads to uncontrolled movements and cognitive dysfunctions. The expansion of huntingtin protein (due to the poly CAG excess mutation and polyglutamine expansion) was found to be the primary cause of cytotoxicity in the striatal and cortical neurons [79].

Despite the large number of studies showing the potent neuroprotective effects of CBD, an early clinical trial showed the limited efficacy of CBD in reducing hyperkinesia (chorea) severity in Huntington’s patients [80]. Nevertheless, the *Sativex* botanical mixture was recently used in malonate-lesioned rats (an inflammatory model of Huntington’s disease), and was shown to reduce striatal neurodegeneration and neuroinflammation, as well as astrogliosis (reduced GFAP increase). This effect was mediated via CB1 and CB2 receptors, pointing to the possible dominating role of THC in the beneficial activities, although CBD-mediated increases in endocannabinoid tone and indirect receptor activation could not be excluded [81].

## 5. Epilepsy

Epileptic disorders include a wide range of CNS pathologies, resulting from either developmental abnormalities (encephalopathies) or accompanying CNS insults including cancer, intoxication, infection, acute injury, neuroinflammation, and neurodegeneration. Due to the heterogeneous background and various severity levels (from impaired consciousness to severe paroxysms), seizures are many times difficult to be treated, being frequently highly resistant to therapeutics. Moreover, in many cases the effective therapeutics cause intolerable side effects.

Astrocytes have been suggested as a potential target for seizure control due to their essential role in neuronal development, activity (neuronal discharges), including glutamate turnover, as well as their close interaction with the brain vascular system. As described above, astrocytes are considered as the main cell type responsible for the uptake of glutamate, which is metabolized later to glutamine that in turn is critical for glutamate and GABA formation inside the cell [82]. The astrocyte-controlled balance between glutamate and GABA is a critical factor in seizure occurrence [83], and is the main target of the current therapeutics.

Indeed, data exists linking glial dysfunction and susceptibility to seizures/epilepsy [84]. *Cannabis*-based preparations have been used for centuries to control seizures [85]. This mostly anecdotal data was to some extent experimentally confirmed for THC and CBD or their combinations as early as the 1970s with inconclusive results. THC was shown to have both pro- or anti-convulsive effects depending on the concentrations used. CBD was documented to exert a potent antiepileptic effect in humans [86] and in preclinical models [87,88,89]. Recent reports suggest that CBD-rich preparations may be effective in reducing drug resistant epilepsies, including rare but devastating childhood epilepsy syndromes such as Dravet syndrome (Epidiolex, GW Pharmaceuticals, Salisbury, UK).

Not much is known about the effects of CBD on astrocytes in epileptic disorders. Anti-epileptic activity of CBD was recently described in rats with chronic pentylenetetrazole-induced seizures [90]. The CBD anti-epileptic effect was accompanied by decreased astrocyte hyperplasia (decrease in GFAP expression) along with decreased neuronal loss and decreased NMDA1 expression in the hippocampus.

## 6. Neuropsychiatric Disorders

Neuropsychiatric disorders are complex medical conditions with diverse and mostly unclear etiologies. Disruptions in neurotransmitter systems, including in their metabolism, release, and uptake, have been considered as the key causes of neuropsychiatric pathologies such as depression, anxiety, and schizophrenia. However, mild, but chronic, neuroinflammation and neurodegenerative processes can contribute to mental/neuropsychiatric disorders via impairing neuroplasticity and synaptic functions. Thus, anti-oxidant and anti-inflammatory drugs were proposed to be therapeutic not only in stroke and age-related neurodegeneration but also in depression and anxiety disorders.

Due to the essential role of astrocytes in modulating synaptic function, neurotransmitter turnover, and neuronal plasticity (the “tripartite synapse” concept), as well as ion and oxidative balance in the brain, astrocytes are considered an important therapeutic target in neuropsychiatric conditions [53,91]. An increasing number of reports indicates that chronic and acute stressors alter astrocyte morphology and the expression of astrocyte specific proteins in brain areas that are known to play a critical role in emotional processing, such as the prefrontal cortex, hippocampus, and amygdala [92].

CBD was shown to exert beneficial effects in several mental and psychiatric disorders, including psychosis, anxiety, and depression, in animal models [19,24,93,94] and in humans [95,96,97].

The group of Guimaraes [98] evaluated the effectiveness of CBD as an anti-psychotic drug using a mouse model of schizophrenia based on the *N*-methyl-d-aspartate (NMDA) receptor hypofunction (chronic administration of MK-801, NMDA antagonist). CBD, indeed, prevented the MK-801-induced cognitive impairments (negative symptom of schizophrenia) and anxiety in the tested mice. These behavioral changes were accompanied by reduced neuroinflammation (microglial Iba1 expression) as well as by reduced astrocytic activity, manifested by reduced GFAP expression in the medial prefrontal cortex [98].

In addition, we would like to note that CBD has been recently shown to diminish heroin self-administration in animals [99], and it may inhibit drug-seeking behavior in humans [100], opening a new path in addiction treatment. The role of activated astrocytes in drug addiction is well-recognized [101], but CBD’s effect on (astro)glial activity in drug addiction and/or dependence has not been addressed so far.

## 7. Neurogenesis

Adult hippocampal neurogenesis is positively associated with brain plasticity, cognitive and emotional functions, buffering stress and stress induced-endocrine responses, and facilitating brain recovery [102,103]. Astrocytes are suggested to have a double-edged role in neurogenesis. Although reactive astrocytes suppress neurogenesis [35,104], astroglial cells were reported to fill neurogenic niches during development and in the adult brain and to support neurogenesis. Subpopulations of parenchymal glia may serve as neural stem/progenitor cells (NSPC) [35] that can differentiate into neurons, astrocytes, and oligodendrocytes, replenishing the cell loss that occurs following injury [105]. Interestingly, mature astrocytes express proteins which are also expressed by NSPC in the adult brain (GFAP, S100β, aldehyde dehydrogenase 1 family member L1, GLAST and GLT1 as well as glycogen granules [35]).

Impaired neurogenesis was shown to result in anxiety-like behaviors and depression [103,106]. Campos and co-authors [107] showed that the anxiolytic effect of CBD in mice subjected to chronic unpredictable stress was accompanied by increases in hippocampal neurogenesis as tracked using GFAP-thymidine kinase (GFAP-TK) transgenic mice. Complementary in vitro studies revealed that CBD promotes hippocampal progenitor cell proliferation and cell cycle progression by increasing endocannabinoid tone and via CB1/CB2 receptors. Interestingly, the effect was lost at higher CBD concentrations [107]. CBD promoted the viability of a nestin-positive stem cell population in cultured whole-brain derived NSPCs, although no changes in GFAP mRNA levels, as an indicator of astrocyte-like differentiation, were observed when these cells were grown in culture without growth factors [108].

## 8. Receptors Involved

Despite growing interest in CBD’s beneficial effects across a variety of CNS threats and disorders, its mechanisms of action are still unclear. Early studies did not find CB receptors in astrocytes [109,110], but later reports indicate that CB1 as well as CB2 receptors are expressed in these cells, and play critical roles in synaptic transmission and inflammatory responses [16,111,112,113,114]. As described earlier, the effect of CBD does not seem to involve directly CB1 and CB2 receptors [29,70,71,115,116]. However, indirect effects of CBD, e.g., via FAAH inhibition and the upregulation of endocannabinoid tone, cannot be excluded, and was indeed shown to be involved in many cases [107,116,117], including neuropsychiatric disorders [96]. In addition, CBD was shown to regulate directly or indirectly the activity of the peroxisome proliferator-activated receptor gamma (PPARγ), serotonin 5-HT_1A_ receptor, the adenosine transporter A_2A_, and selective members of the transient receptor potential vanilloid (TRPV) family, both in adult and the developing brain [26,45,58,74,118]. The question if any of these targets is involved in the modulation of astrocyte activity by CBD is still open.

## 9. Remarks

### 9.1. Serum-Free Sensitization to CBD Toxicity

CBD was found to be cytotoxic in human cancer glioblastoma multiforme cell lines [119,120,121], but to not affect the cell viability of primary, human, non-transformed astrocyte cells [121]. Similarly, THC was shown to be cytotoxic in rat glioma cells, but not in primary rat newborn astrocyte cultures unless serum deprivation (i.e., serum defined medium) was applied, which resulted in extensive cell death in the presence of THC [122]. CBD toxicity under no serum conditions was described by our group in a transformed microglial BV-2 cell line [123], suggesting a generalized CBD activity under low nutrient conditions. Serum withdrawal is occasionally used to study the proinflammatory role of primary astrocytes to augment cytokine release [124]. Above observations indicate that serum-free conditions should be avoided when using cannabinoids to study changes in cytokine levels that may be nonspecifically affected by reduced cell viability.

### 9.2. Astrocyte Activity Markers

**Figure 1:**
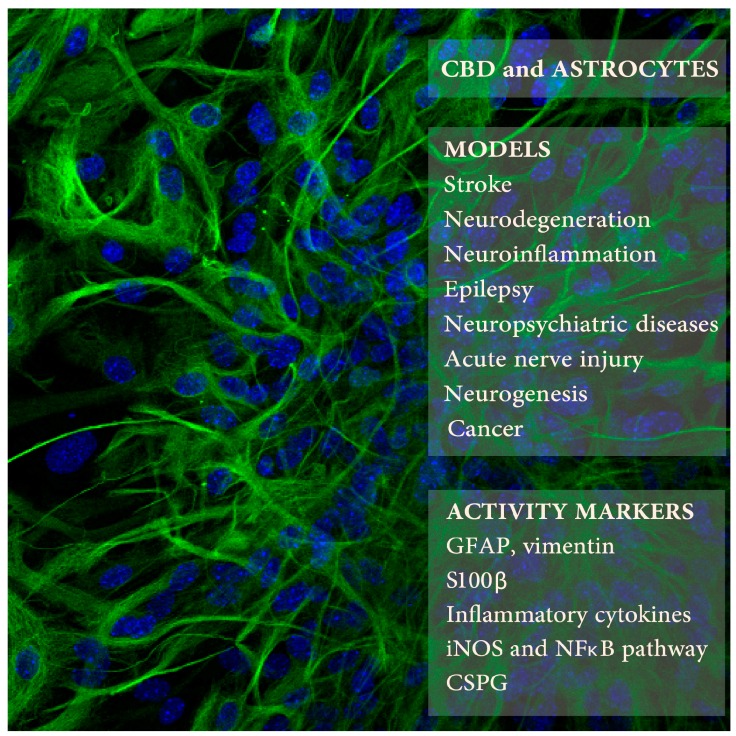
Cannabidiol (CBD) effects on astrocytes was studied in several animal models of neuropathologies (upper right frame) using selected markers of astrocyte activity (lower frame). The background picture presents mouse astrocytes in culture expressing glial fibrillary acidic protein (GFAP) (in green; blue color indicates cell nuclei; Kozela, Juknat, and Vogel, unpublished data).

GFAP expression is the main astrocyte activity marker used in all of the reports listed here, across various species, models, and developmental stages (Figure 1). GFAP is the major intermediate filament (IF) protein in astrocytes. Although changes in GFAP levels remain the universal indicator of astrocyte activation [125], one has to remember that its levels vary in brain areas and across species in various developmental phases. GFAP is the major IF protein in mature astrocytes while vimentin is dominant in the neonatal brain. GFAP has been recognized as a marker for NSPC lineages. Thus, increased GFAP expression may serve as an indication of reactive gliosis as well as astrocyte maturation, brain aging, or neurogenesis as a sign of brain repair [125]. Using complement markers may help to achieve a more accurate description of astrocyte activity.

### 9.3. Astrocyte Heterogeneity

Research in the last decade has revealed enormous heterogeneity in astrocyte populations, even within the same brain areas, including in their morphology, differences in functional gap junctional coupling, and the expression of transmitter receptors, membrane currents, and glutamate transporters [126]. Reactive astrocytes were recently characterized based on their distinct gene expression set ups, representing either a proinflammatory A1 subtype promoting lipopolysaccharide (LPS)-induced neuroinflammation, or an anti-inflammatory A2 phenotype that has a protective role after ischemia [127,128]. Similar functional diversities apply to microglial cells, defined as M1 and M2 phenotypes [129], and point to the substantial plasticity of glial cells. Moreover, despite evidence for the negative role of reactive gliosis, astrocytes have well-established roles as neuroprotective, angiogenic, immunomodulatory, neurogenic, or antioxidant depending on the disease or on its phase.

Interestingly, CBD is well known to dampen various inflammatory responses, but it was recently shown to be a potent activator of several endogenous regulatory mechanisms. We and others have shown that CBD upregulates the anti-inflammatory cytokine IL-10 [71,78,130], the immunoregulatory genes in autoimmune T cells mediating EAE/MS [72,73], and induces inhibitory cell phenotypes resolving inflammation, including myeloid-derived suppressor CD11b+Gr1+ cells [131] and CD4+CD25+LAG3+ T cells [72]. Thus, boosting protective astrocyte activity, including astrocyte-mediated neurogenesis, might be an attractive therapeutic approach and CBD may offer such a possibility.

### 9.4. The Interaction of CBD with Other CNS Cells

Although a majority of data indicates that activated astrocytes are silenced or modulated in the presence of CBD, a direct interaction of the drug with these cells has not been sufficiently evidenced yet. Based on the collected data, it is not clear if the diminished astrocyte activity described to date results from direct effect of CBD on the astroglia, or rather whether it is a consequence of previously CBD-improved neuronal or oligodendrocyte viability [25,70], and decreased microglial activity [29,30,70], which altogether results in reduced reactive gliosis. The precise sequela of the CBD rescuing effects in the CNS pathology, as well as the interaction of astrocytes with other CNS resident cells in the presence of CBD, is still there to be addressed.

## 10. Conclusions

CBD was shown to be potentially therapeutic across a wide spectrum of diverse neuronal disorders (Table 1). Such a broad activity of CBD points to possible common etiology/mechanisms. Indeed, disturbed oxidoreductive homeostasis, impaired debris cleavage, or chronic neuroinflammatory processes were shown to be the leading causes of neurodegeneration, to impair recovery from brain injury as well as to contribute to cognitive and neuropsychiatric deficiencies. CBD combines potent anti-oxidant, anti-inflammatory, and neuroprotective properties. Astrocytes may serve as an important target for CBD activity, due to their significant role in maintaining ion and metabolic balance, debris cleavage, and proper neuronal activity. Although many studies indicate that activated astrocytes are modulated in the presence of CBD, a direct binding of CBD to astrocytes has not yet been observed. Moreover, the mechanisms by which CBD facilitates brain recovery via endorsing protective astrocyte function(s) is still there to be explored.

## Figures and Tables

**Table 1:** CBD (Cannabidiol) modulation of astrocyte activity in neuropathological models. If multiple doses/time points of CBD application are presented, the effective doses/time points are bolded.

CBD Doses/Times	Animals or Cells	Procedure	Astrocyte Activity Measures	Other CBD Activities	Refs.
**Hypoxia-Ischemia**					
0.1, **1, 3 mg/kg** i.p. just before and 3 h after the MCAO occlusion	ddY mice, 25–35 g	Middle cerebral artery occlusion (MCAO), 4 h	Day 3: decreased GFAP IHC (infarct including striatum)	Decreased infarct size, reduced microglia activation and apoptosis, improved neurological score, motor coordination; Infarct size reduction—not CB1 and CB2 mediated	[41]
3, **10, and 30 mg/kg** i.p., 30 min before, 3, 24, 48 h after BCCAO	Swiss mice, 35–45 days old (30–40 g)	Bilateral common carotid artery occlusion (BCCAO) using aneurysm clips, 17 min	Day 7: decreased GFAP IHC in hippocampus (HPC)	Reduced neuronal cell death and improved spatial learning	[42]
1 mg/kg s.c. **10 min, 1, 3, 6, 12, 18**, 24 h after HI	C57BL6 mice, 9–10 days old	Left common carotid artery electrocoagulation, 3 h later followed by hypoxia (10% O_2_) for 90 min	Day 7: decreased GFAP level (astrocyte viability)	Reduced ipsilateral hemisphere volume loss and microglia activation	[43]
5 mg/kg i.p., 15/30 min after MCAO	Wistar rat pups, 7–9 days old	MCA occlusion, 3 h	Day 7: no effect on GFAP; Day 15: reduced GFAP (parieto-occipital cortex)	Improved neurobehavioral scores, reduced neuronal damage and microglia activation, no effect on infarct size	[44]
1 mL of 0.1 mg/kg i.v. per ~1.7 kg weight, **15, 240 min** after the HI	Piglets, 1–3 days old	Clamping both carotid arteries with vascular occluders and low oxygen (8–10%) for 20 min	72 h post HI: CBD reversed astrocyte loss and morphology (GFAP IHC, less swallen), decreased HI-elevated S100β in the CSF	Reduced neuronal and astrocytic cell death, less TNFα(+) cells, improved brain activity and neurobehavioral performance	[45]
100 nM, 1 and **10 μM** before or immediately after the OGD, and 2 and 4 h into reperfusion	Human brain microvascular endothelial cell (HBMEC) and human astrocyte (HA) co-cultures (BBB model)	Oxygen-glucose deprivation (OGD), 4 h	4–32 h post OGD: improved BBB permeability; 32 h post OGD: decreased cell damage (LDH release) and VCAM1 (ELISA); minor but significant decreases in IL-6 and VEGF (but not of IFNγ, IL-10, IL-1β, IL-2, CCL3, CCL4, or TNFα)	Protective effect up to 2 h into reperfusion; PPARγ and partially 5-HT_1A_ mediated (not via CB1, CB2, TRPV1, A_2A_) Monocultures *HBMEC*: CBD increased IL-6, VEGF but decreased VCAM1; *HA*: CBD decreased VCAM1	[47]
**Neurodegeneration**					
5, **15, 30 mg/kg** i.p., daily for 5 days post lesion	Wistar rat pups, 2 days old	Unilateral sciatic nerve transection at mid-thigh	Day 5: decreased GFAP IHC (only 15 mg/mL CBD analyzed) in ventral horn of the lumbar spinal cords	CBD 15 mg/mL rescued synaptic and sensory neurons losses, reduces microglia activation	[50]
**2.5 or 10 mg/kg** i.p., daily for 7 days starting day 3 after Aβ	C57BL/6J mice, 3–5 month old	Human Aβ (1–42, 10 ng/mL) inoculation into the right dorsal hippocampus (HPC)	Day 10: decreased GFAP mRNA (in situ) and protein IHC in HPC	Decreased iNOS and IL-1β levels	[57]
10 mg/kg i.p., for 15 days	Sprague-Dawley (SD) rats, 300–350 g	Human Aβ (1–42; 30 ng) into HPC CA1	Day 15: Decreased GFAP, S100β in HPC homogenates and GFAP IHC in HPC CA1	Decreased neuronal damage, neuroinflammatory signaling, increased calbindin levels in HPC CA1 and neurogenesis in the HPC DG; PPARγ mediated	[58]
10^−9^–10^−7^ M	Cultured newborn SD rat astrocytes	Aβ (1–42) 1 µg/mL	24 h: inhibition of S100β, NO, TNFα, IL-1β release (ELISA) and GFAP, S100β, iNOS, NF-κB (p-p50/p65) levels (WB)	PPARγ mediated	[58]
**1, 5 μM**	Cultured newborn Wistar rat astrocytes	IL-1β + TNFα (both 10 ng/mL); serum free	6 h: decreased CCL-2 (ELISA)	7 days of CBD 5 mg/kg i.p. ameliorated TMEV EAE, decreased leukocyte infiltration, VCAM1, CCL2, CCL5, CCR2 in the PFC; reduced microglial Iba1, TNFα, IL-1β; A_2A_ mediated	[74]
1% CBD in propylene glycol on hind limbs daily post immunization from days 14 (EAE onset)-28	C57BL/6 mice, 12 week old (males)	MOG35–55-induced EAE	Day 28: decreased GFAP IHC and WB in the spinal cords	Diminished clinical EAE score, T cell infiltration and demyelination in the spinal cord, decreased TNFα, IL-6, TGFβ, oxidative markers and apoptosis, increased IL-10	[78]
*Sativex*-like botanical extracts *—10 mg/kg i.p. daily days 70–80 post virus injection	SJL/J mice, 4 week old, (females)	TMEV-induced EAE	Day 80: reduced GFAP and vimentin, CSPG (CS56) IHC, brevican mRNA in spinal cord	Improved motor deficits, decreased myelin and axon damage, T cell infiltration, ICAM1, microglial Iba1, IL-1β, TNFα, IFNγ and increased Arg1 and IL10; Δ9-THC-BDS or Δ9-CBD-BDS alone mimicked *Sativex*-like mix in EAE via CB1 and PPARγ, respectively	[75]
100 nM, **0.5 and 1 μM**	Cultured postnatal Wistar rat astrocytes	TGFβ1 + βFGF (both 10 ng/mL) 24, 48 or 72 h; cultured 1 h in no serum DMEM before stimulation	24 h: reduced brevican and XT-I mRNA. 48 h and 72 h: reduced neurocan (IHC and WB on supernantants)		[75]
3 mg/kg of Sativex-like botanical extracts * i.p., 30 min before and 2 h after injection	SD rats, 12 weeks old	Intrastriatal malonate induced Huntington-like neurodegeneration	48 h: decreased GFAP IHC in striatum	Decreased striatal edema, microglial Iba1, iNOS and IGF1, minor prevention of cell death, reversed malonate-induced CB1 decrease; CB1 and CB2 mediated	[81]
**Other**					
10, 20, **50 mg/kg** 1 h before each PTZ	SD rats, 170 g	Chronic epilepsy, i.p. PTZ for 28 days	Day 28: reduced astrocyte hyperplasia (GFAP IHC in HPC CA1, CA3)	Antiepileptic, decreased neuronal loss and NMDAR1 in the HPC	[90]
30 and **60 mg/kg** i.p., days 6–28 of MK-801 injections	C57BL/6J mice, 6 weeks old	Schizophrenia model based on NMDA receptor hypofunction, 28 days of 1 mg/kg MK-801	Day 31: Slight decrease of GFAP IHC in mPFC	Improved cognitive scores and reduced anxiety, decreased microglial Iba-1	[98]
30 mg/kg i.p. 2 h after each stressor	Wild type and GFAP-TK mice, 3 months old	Chronic unpredictable stress (CUS, 14 days), model of depression/anhedonia	Day 15: In WT increased HPC neurogenesis, including non-stressed controls, reversed CUS-decreased neurogenesis (NeuN, BrdU, and Dcx)	Day 14 and 15—decreased anxiety; effect ablated in GFAP-TK/ganciclovir mice; CB1 mediated, AEA increased (not 2-AG or PEA)	[107]
50, **100, 250**, 500 **nM**	HiB5 hippocampal progenitor cell line	BrdU expression, cell number	Increased BrdU and S phase cell cycle	CB1/CB2 mediated	[107]
1 µM	8-week old mouse whole brain neural/stem progenitor cells (NSPCs)	Whole brain NSPCs in vitro proliferation and differentiation into neurons or astrocytes	Day 2: Increase in nestin mRNA (B27 supplemented medium) and cell viability, no effect on GFAP mRNA	No effect on nestin in complete medium	[108]
10 µM 1–3 days	Human glioblastoma multiforme cells (U87MG, MZC)	Cell death and viability, colony formation following CBD alone and in combination with BCNU, TMZ, and DOXO	Days 1–3: potentiates cytotoxicity of BCNU, TMZ, and DOXO chemotherapeutics	TRPV2-dependent Ca^2+^ influx	[121]
10 µM 1–3 days	Normal human astrocytes (NHA)	Cell death and viability following CBD alone and in combination with BCNU, TMZ, and DOXO	No effect on cell viability alone or in combination with BCNU, TMZ, and DOXO chemotherapeutics		[121]

Abbreviations: BBB, blood brain barrier; CSF, cerebrospinal fluid; CUS, Chronic unpredictable stress; DG, dentate gyrus of the HPC; GFAP, glial fibrillary acidic protein; HA, human astrocyte; HBMEC, Human brain microvascular endothelial cell; HI, hypoxia-ischemia; HPC, hippocampus; IHC, immunohistochemistry; MOG35–55, myelin oligodendrocyte glycoprotein 35–55; NSPC, neural/stem progenitor cells; PFC, prefrontal cortex; PTZ, pentylenetetrazole; TMEV, Theiler’s murine encephalomyelitis virus; WB, Western blotting; WT, wild type; * *Sativex*-like, mixture of Δ9-THC botanical drug substance (Δ9-THC-BDS; 67.1% Δ9-THC, 0.3% CBD, 0.9% cannabigerol, 0.9% cannabichromene and 1.9% other phytocannabinoids) and CBD-BDS (64.8%, 2.3%, 1.1%, 3.0%, 1.5%, respectively).
